# Intelligent diagnosis of lung nodule images based on machine learning in the context of lung teaching

**DOI:** 10.1097/MD.0000000000037266

**Published:** 2024-03-08

**Authors:** Miaomiao Li, Lilei Zhuang, Sheng Hu, Li Sun, Yangxiang Liu, Zhengwei Dou, Tao Jiang

**Affiliations:** a Department of Respiratory and Critical Care Medicine, The Fourth Affiliated Hospital Zhejiang University School of Medicine, Yiwu, Zhejiang, People’s Republic of China; b Department of Gastroenterology, Yiwu Central Hospital, Yiwu, Zhejiang, People’s Republic of China; c Department of Radiology, The Fourth Affiliated Hospital Zhejiang University School of Medicine, Yiwu, Zhejiang, People’s Republic of China.

**Keywords:** imaging, intelligent diagnosis, lung nodules, lung teaching, machine learning

## Abstract

The vast majority of intelligent diagnosis models have widespread problems, which seriously affect the medical staff judgment of patients’ injuries. So depending on the situation, you need to use different algorithms, The study suggests a model for intelligent diagnosis of lung nodule images based on machine learning, and a support vector machine-based machine learning algorithm is selected. In order to improve the diagnostic accuracy of intelligent diagnosis of lung nodule images as well as the diagnostic model of lung nodule images. The objectives are broken down into algorithm determination and model construction, and the proposed optimized model is solved using machine learning techniques in order to achieve the original algorithm selected for intelligent diagnosis of lung nodule photos. The validation findings demonstrated that dimensionality reduction of the features produced 17 × 1120 and 17 × 2980 non-node matrices with 1216 nodes and 3407 non-nodes in 17 features. The support vector machine classification method has more benefits in terms of accuracy, sensitivity, and specificity when compared to other classification methods. Since there were some anomalies among both benign and malignant tumors and no discernible difference between them, the distribution of median values revealed that the data was symmetrical in terms of texture and gray scale. Non-small nodules can be identified from benign nodules, but more training is needed to separate them from the other 2 types. Pulmonary nodules are a common disease. MN are distinct from the other 2 types, non-small nodules and benign small nodules, which require further training to differentiate. This has great practical value in teaching practice. Therefore, building a machine learning-based intelligent diagnostic model for pulmonary nodules is of significant importance in helping to solve medical imaging diagnostic problems.

## 1. Introduction

It is crucial to perform early diagnosis of lung nodules (LN) for early detection and treatment of lung cancer as the incidence of lung cancer has been rising annually in recent years due to rapid socioeconomic development, environmental pollution, smoking, and unreasonable lifestyles.^[1,2]^ The most efficient way to diagnose LN images and look for the best solution is to use evolutionary optimization methods, but they typically have issues with incomplete feature search and subtle differentiation of benign and malignant nodes. This is due to the difficulty of performing intelligent diagnosis (ID) in LN images and the difficulty of accurately analyzing the target in time. With the aid of objective optimization equations, different kinds of machine learning (ML) models, particle swarm methods, and ID for LN images are examined and put into practise.^[3]^ The bulk of ID models now available, however, have substantial zone distribution and incomplete feature capture, which has a considerable negative impact on how well healthcare providers are able to assess patient damage. In order to target the initial algorithm chosen, the data are classified into Benign nodules (BN), Malignant nodules (MN), and Non-nodular (NN) by LN type, and optimized according to the optimization model with LN image ID as the objective. As a result, different algorithms must be used depending on the situation. The algorithm employs real patient data for analysis, the optimized ML algorithm, the pertinent mathematical algorithm and optimization conditions, and the solution of the suggested model to ultimately achieve efficient diagnosis.^[4,5]^ The study will be divided into 4 sections: an overview of the ML-based LN image ID algorithm in the first section, a study of the ML-based LN image ID model in the second section, an experimental validation of the second section in the third section, and a conclusion and discussion of the research drawbacks in the fourth section.

## 2. Related works

Deep learning algorithms and neural network algorithms have been used in various industries recently, but many professionals and academics are currently concentrating their studies on LN image intelligence technology. The ongoing advancement of computer and image processing technologies has given the study of medical image intelligence good technical support. Through computer-aided software for image processing and analysis, automatic identification, segmentation, annotation, and classification of LNs in CT images can be accomplished in LN diagnosis. Deep learning algorithms and neural network algorithms can be used to automatically detect, segment, and classify LNs, considerably enhancing the effectiveness of medical professionals. The primary goal of the investigation is to better understand LN image ID and to apply the results to the setting of lung instruction. A novel deep learning-based strategy for LN image recognition is proposed to be studied by Ather S et al The method was examined for graded LN monitoring and tracking data and produced useful application results. According to the experimental findings, the algorithm has broad applicability and can be used to create and assess radiologists’ approaches.^[6]^ By enabling efficient segmentation and risk assessment of stroke imaging data, Zhu Guangming and other researchers discovered that artificial intelligence technology plays a very significant role in stroke imaging research. The study findings demonstrated that deep learning-based imaging technology can analyze and forecast image data, leading to the development of a systematic overview of deep learning-based stroke imaging technology.^[7]^ Lima Since most conventional diagnostic techniques rely on LN pictures, which makes diagnosis highly challenging for physicians, Lucas L research team discovered that using a mix of simulated annealing and tree estimation could increase diagnostic precision. The experimental findings revealed that the new model significantly increased the accuracy of image analysis for many LNs, raising hope for a greater number of LN patients’ survival.^[8]^ Varela-Santos S et al concentrate on local binary texture characteristics, gray-scale co-occurrence matrices, and gray-scale histograms using Second, to create the best neuro-fuzzy classifier, the chest film lesions were feature-shortened using a multi-objective genetic algorithm. Based on this, the photos are feature-segmented using a modular neural network, allowing for in-depth analysis of the images. Testing on 3 sets of substantial chest X-ray film samples allowed researchers to confirm the algorithm efficiency.^[9]^ A non-linear mapping of radial baseline features is created by the research team of Zhang Y using an image classifier that is built on feature correlation analysis. Utilizing both collaborative hospital data and publicly accessible data, the proposed method will be examined. The experimental findings show that for edge-unstable nodes, the model offers a significant improvement in sensitivity, specificity, and accuracy. In addition, our strategy outperformed previous deep learning diagnostic approaches in terms of discriminative outcomes, and it is ideal for LN diagnosis.^[10]^

In conclusion, a variety of academics and researchers have made contributions to image ID and ML. In order to meet more effective data set processing and optimization techniques, many better algorithms have been developed. The method is also utilized to optimize the ID of LN pictures, which should have no less significance in the medical industry given the better data processing capability of the ML model and the current shortcomings in conventional diagnosis.

## 3. Diagnostic model for incorporating ML in LN imaging

To facilitate the development of LT, a Support Vector Machine (SVM) based diagnostic model for LN imaging is proposed. The proposed scheme is to apply SVM techniques to the teaching of LN image ID. Firstly, based on SVM delving into Particle Swarm Optimization (PSO), combined with Random Forest algorithm, allows the proposed ML algorithm to be used to help healthcare professionals diagnose a patient condition through the ID of an image.

### 3.1. LN imaging diagnostic model incorporating SVM

Although the adaptive threshold-based nodal segmentation method is simple and efficient, there is also a large number of NNs that are mis-segmented, when a direct use of the SVM classifier would result in excessive computational effort and low diagnostic efficiency and accuracy. To solve this problem, a new method are adopted to perform feature cropping for each candidate node, which allows significant nodeless objects to be cropped out. The rule-based cropping method focuses on cropping based on features such as mean, variance, circle, flat, slender, and rectangle. A threshold is set and when this value is greater or less than this threshold, the sample is identified as a knot or a knotless. Part of the knotless feature set is first eliminated to reduce the false alarm rate, and then the remaining feature set is used as the feature set of SVM for classification detection.^[11,12]^ SVM is a supervised ML method based on kernel functions, which uses kernel functions to transform the samples so as to predict the optimal boundary between 2 classes. Therefore, it finds a way to differentiate the data based on the defined labels through a complex data transformation. To obtain the optimal classification plane, as shown in Equation (1).


$$\begin{array}{l} \left\{ \begin{array}{l} w^{T}t_{i}+b\geq 1,g_{i}=1\;\\ w^{T}t_{i}+b\leq -1,g_{i}=-1\; \end{array}\right. \end{array}$$ (1)

In Equation (1), when $w^{T}t_{i}+b=0$, which is the optimal plane, 1 represents the nodal label and −1 is the NN label. In order to maximize the distance between the nodule and the NN, as shown in Equation (2).


$$\begin{array}{l} \max\frac{2}{∥w∥}\Rightarrow \min\frac{1}{2}{∥w∥}^{2} \end{array}$$ (2)

In Equation (2), $\max\frac{2}{∥w∥}$ is the classification plane of the nodule and $\min\frac{1}{2}{∥w∥}^{2}$ is the maximum distance between the nodule and NN. The SVM model for quadratic programming is defined in terms of the generalized Lagrangian function, as shown in Equation (3).


$$\begin{array}{l} g\left(x\right)=sgn\left(w^{*T}x+b^{*}\right) \end{array}$$ (3)

In Equation (3), $b^{*}$ and $w^{*}$ are the weight vectors. Commonly used kernel function forms are linear kernel, radial basis function kernel, polynomial kernel and S-shaped kernel, using the RBF kernel function as shown in Equation (4).


$$\begin{array}{l} K\left(x_{1},x_{2}\right)=\exp\left(-\gamma {∥x_{1}-x_{2}∥}^{2}\right) \end{array}$$ (4)

**Figure 1.**
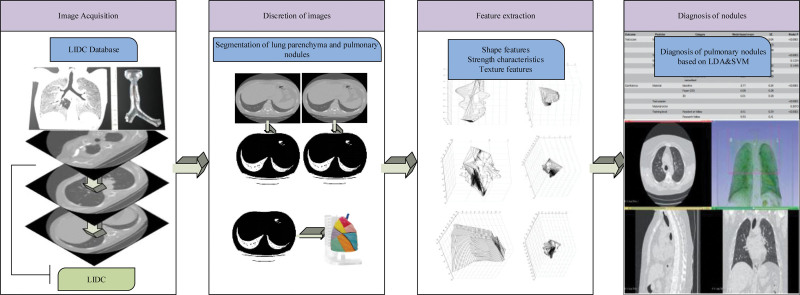
The proposed automatic detection process for pulmonary nodules.

In Equation (4), $CA_{x}$ is the parameter and $K\left(x_{1},x_{2}\right)$ is the RBF kernel function. The implementation process of the LN diagnosis method based on this type of hybrid feature and SVM is shown in Figure 1.

In Figure 1, the LN is identified using image data in DICOM format from the LIDC-IDRI database as an example. Based on this, an adaptive segmentation method based on a combination of cavity filling and rolling spherical shape was proposed based on the characteristics of the CT images, and the final threshold was selected. Based on this, the mean offset method was used to remove noise and the adaptive threshold method was used to preprocess the image for LN. At the same time, a mixture of features containing gray scale, shape, texture and other features was extracted so that it could better reflect the feature information of the candidate nodes. Finally, in terms of node recognition, a hybrid classifier based on a combination of SVM and rules is proposed in this paper in order to overcome the shortcomings that exist in traditional single classifiers. In complex clinical scenarios, the effectiveness of diagnosis and treatment may become exceptionally complex depending on the population (such as age, gender, genetics, lifestyle, disease history, etc.). For example, for patients with rare diseases, atypical symptoms, or multiple diseases at the same time, the diagnostic process may be extremely complex and difficult to carry out. In complex research designs, such as multicenter clinical trials, data collection and processing may face many challenges. Sometimes, patients may not be able to accurately treat according to medical advice, which may lead to uncertainty in treatment outcomes. Different brands or models of equipment may lead to differences in test results, making diagnosis and efficacy evaluation difficult. In medical image analysis, the performance of scanning equipment, the choice of technical means, the influence of patient position, respiration or pulse, and even differences in software versions may affect the quality of images, thereby affecting the analysis results. In order to demonstrate the effectiveness of the method and overcome the aforementioned uncertainty, a mixed dataset was attempted, and adjustments were made to the resolution, contrast, brightness, hue, and other aspects of the image. It was found that even in such complex situations, the method can still perform well in recognition. To further demonstrate this point, an evaluation was conducted from the perspective of image quality. Structural similarity index (SSIM) and peak signal-to-noise ratio (PSNR) were used as evaluation indicators, and the image was denoised. It was found that even after various adjustments were made to the image, the method showed excellent performance in both SSIM and PSNR, further confirming the effectiveness and robustness of the method in processing complex medical images. It does not deny the uncertainty of the method, but through empirical research and in-depth analysis, it has been proven that the method can still maintain efficiency and accuracy in the face of complex clinical scenarios and data challenges. This not only reflects the superiority of the method, but also reflects a rigorous scientific attitude and determination to work hard to solve problems in the research process. The use of mixed datasets, various image adjustments, and the evaluation of SSIM and PSNR have fully demonstrated the effectiveness and robustness of the proposed method in dealing with complex clinical scenarios and data challenges. In this process, a reasonable construction and parameter setting of the learning model, as well as the selection of training samples and the validation of the classifier, are required. While defining the probability density is directly related to the gray scale value of the pixel points, the total probability density equation, as shown in Equation (5).


$$\begin{array}{l} {K_{h_{s}}}_{h_{r}}\left(x\right)=\frac{C}{h_{s}^{2}h_{r}^{2}}K\left({∥\frac{x^{s}-x_{i}^{s}}{h_{s}}∥}^{2}\right)K\left({∥\frac{x^{s}-x_{i}^{s}}{h_{r}}∥}^{2}\right) \end{array}$$ (5)

**Figure 2.**
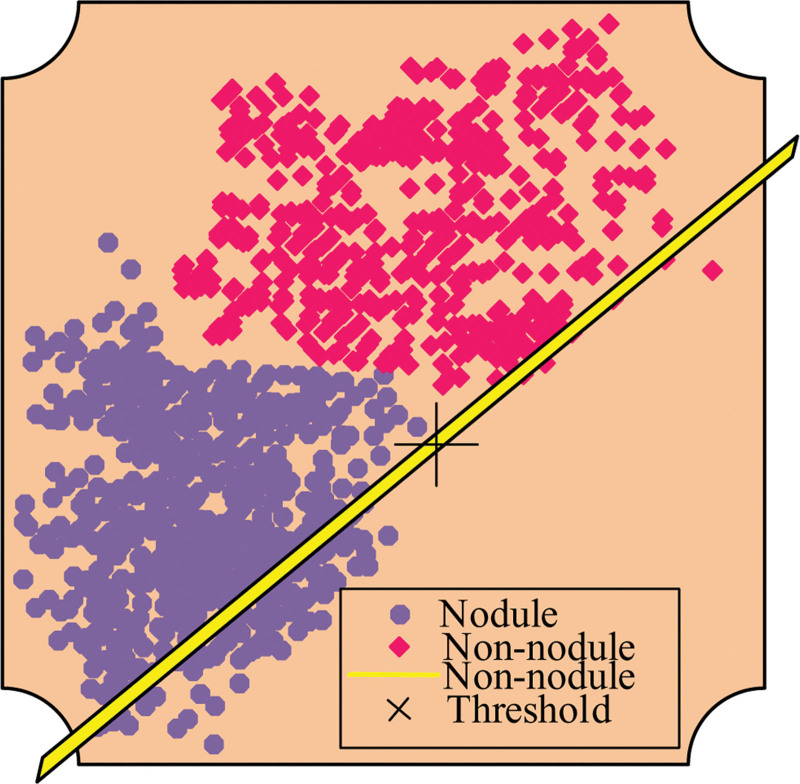
LDA transform features and projections. LDA = linear discriminant analysis.

In Equation (5), $K\left({∥\frac{x^{s}-x_{i}^{s}}{h_{s}}∥}^{2}\right)$ is the spatial location information, $K\left({∥\frac{x^{s}-x_{i}^{s}}{h_{s}}∥}^{2}\right)$ is the gray value information, and $x_{i}$ A is the pixel point. Since high-dimensional hybrid features have characteristics such as noise and non-linearity, which make the identification of nodes difficult, it is necessary to carry out feature selection on them. In general, the basic idea of feature selection is to firstly select the categories that have an important impact on the detection results and eliminate the unwanted categories; secondly, to dimensionally approximate the Govett collection data so that it retains the original extracted feature information. The first method is simple, but it leads to the loss of feature information, which affects the effectiveness of fault diagnosis, therefore, the second method is proposed in this project to reduce the dimensionality of the features. Principal Component Analysis (PCA) and Linear Discriminant Analysis (LDA) are 2 commonly used methods for data dimensionality reduction, the difference being that PCA is an unsupervised method that requires no training and LDA is a supervised method that requires labels to be provided to the data. lda is a method that requires labels to be added to the data supervised method.^[13,14]^ the LDA algorithm not only guarantees the optimal score, but also maintains the maximum information of the data sample, as shown in Figure 2.

In Figure 2, LDA reduces the computational overhead of detection by mapping the feature space on the dataset to a lower dimensional space, while retaining the ability to discriminate between dissimilar samples, and also reduces errors in parameter estimation and avoids overfitting.The basic assumption of LDA is that the distribution of data within each category follows a Gaussian distribution. The main goal of LDA is to find a linear combination method that enables different categories of data to be clearly distinguished on one or more dimensions. It achieves this goal by minimizing intra group variance and maximizing inter group variance. Unlike variance based dimensionality reduction methods such as principal component analysis (PCA), LDA explicitly considers category information. Although LDA is a dimensionality reduction technique, it is often used in preprocessing steps to improve the performance of other machine learning models, such as SVM. When processing high-dimensional data, the advantages of LDA include reducing overfitting risks, increasing computational efficiency, improving data visualization, and improving learning efficiency. In this way, the SVM algorithm can find better hyperplanes to segment data of different categories during training, thereby improving its accuracy. However, it is worth noting that the effectiveness of LDA is influenced by the linear separability and normal distribution assumptions of the data, and LDA processing may not yield ideal results for data that violate these assumptions. In addition, the high dimension, small sample size problem is also a concern for LDA, so in practical applications, methods need to be selected and adjusted according to the specific situation of the data.

### 3.2. LN image construction incorporating logistic regression (LR)

**Figure 3.**
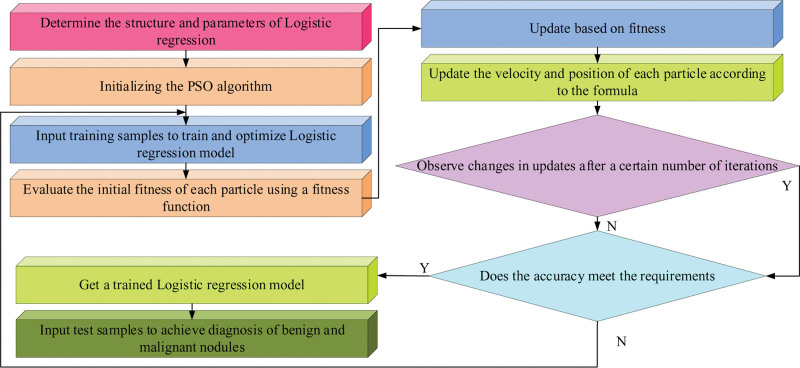
Diagnosis of benign and malignant pulmonary nodules based on LR. LR = logistic regression.

The study was approved by the Ethics Committee of The Fourth Affiliated Hospital Zhejiang University School of Medicine. PSO is an evolutionary computing technique based on swarm intelligence, on the basis of which an algorithm based on parameters such as inertia weights, cognitive learning factors and social learning factors is proposed to achieve a fast correction of particle motion trajectories. At each iteration, all particles are evaluated according to the fitness and the best fitness will be obtained, iteratively, until the value of the fitness is equal to the specified value, or the cycle is completed with the default value. Based on this, the PSO-LR classifier was constructed using PSO to optimize the LR classification parameters to discriminate between benign and malignant LNs.^[15]^ The flow of nodule diagnostic classification using LR is shown in Figure 3.

In Figure 3, LR is a statistical method that is widely used in dichotomous problems and is used to solve the diagnosis of benign and malignant nodules. In regression analysis, the 2 basic methods for parameter estimation are least squares and maximum likelihood estimation. maximum likelihood estimation is mainly used in LR, where a likelihood function needs to be established before estimating the parameters, as shown in Equation (6).


$$\begin{array}{l} p=\frac{e^{\alpha +\sum_{j=1}^{k}\beta_{i}x_{i}}}{1+e^{\alpha +\sum_{j=1}^{k}\beta_{i}x_{i}}} \end{array}$$ (6)

In Equation (6), i is the number of particles, and the log-likelihood function of the LR model is taken as the fitness function for each particle in the evaluation population in Equation (7) when optimizing the parameters by training samples and the PSO algorithm.


$$\begin{array}{l} J\left(\theta \right)=\sum_{i=1}^{n}\left[y_{i}\left(\alpha +\sum_{j=1}^{k}\beta_{j}x_{ij}\right)-\ln\left(1+e^{\alpha +\sum_{j=1}^{k}\beta_{i}x_{i}}\right)\right] \end{array}$$  (7)

In Equation (7), for the problem of multi-dimensional search space, the position of the i th particle is $X_{i}=\left[x_{i1},x_{i2},\cdots ,x_{iD}\right]$, and the velocity of the i th particle, as shown in Equation (8).


$$\begin{array}{l} v_{id}^{new}=w\times v_{id}^{old}+c_{1}r_{1}\left(\;pb_{id}^{old}-x_{id}^{old}\right)+c_{2}r_{2}\left(gb_{d}^{old}-x_{id}^{old}\right) \end{array}$$  (8)

in Equation (8), while the velocity vector of the i th particle is $V_{i}=\left[v_{i1},v_{i2},\cdots ,v_{iD}\right]$, $PB_{i}$ is the previous best position for the best fitness value of the ith particle, and $GB_{i}$ is the best position of the ith particle so far.^[16]^ The inertia weights are calculated as shown in Equation (9).


$$\begin{array}{l} w=w_{\max}-\frac{w_{\max}-w_{\min}}{iter_{\max}}\cdot iter \end{array}$$ (9)

**Figure 4.**
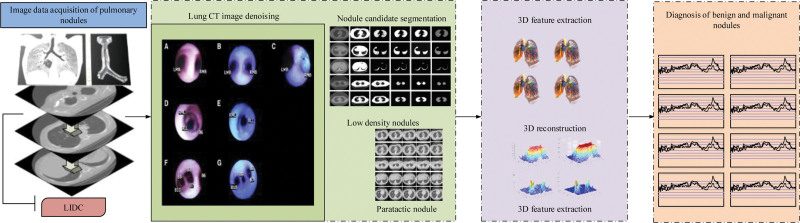
Diagnosis process for benign and MN. MN = Malignant nodules.

In Equation (9), w is the inertia weight and the global optimal solution is $w_{\max}$, while $w_{\min}$ tends to local search. $w_{\min}$ and $iter_{\max}$ are the current and maximum number of iterations, respectively. meanwhile, the LN benign and malignant automatic diagnosis method based on geometric activity contour and LR is proposed.^[17]^ The implementation process mainly includes 4 parts: image acquisition, image denoising and segmentation, LN 3D feature extraction and benign and malignant classification and diagnosis, as shown in Figure 4.

In Figure 4, the diagnostic process of LN benignity and malignancy mainly consists of a cT lung sequence image selected from the database, which is not suitable for image segmentation and detection due to the high noise level. Then, based on the idea of active contour map segmentation, the energy function was improved by combining the information from both inner and outer regions to achieve the segmentation of small lung nodule candidates. Further, for more accurate determination of LN benignity and malignancy, a 3D reconstruction algorithm with gray scale, shape and texture as the main features was investigated to construct features containing 3-dimensional information, a streamlined sample set and with higher accuracy for LN benignity and malignancy. Finally, an efficient classifier of PSO-LR was constructed to solve the problem of quantitative analysis of benign and malignant lung nodules, and classification and diagnosis were performed.

### 3.3. Incorporating random forest LN3D feature extraction and classifier construction

As pre-defined segmentation contour lines with artificially set seed points generate a high number of NNs, the identification of NNs, benign and malignant nodules needs to be increased when performing 3D reconstruction. Five grayscale and texture features were re-extracted. The complementarity of the gray levels allows for the extraction of energy, moisture, and calcification levels. Energy and entropy values are 2 important features reflecting the local homogeneity of the image, and the usual nodules have higher energy and lower entropy values, thus allowing them to be distinguished from NN. The expression for the energy of a nodule, as shown in Equation (10)


$$\begin{array}{l} E_{x}=\sum_{k=0}^{L-1}{\left[H\left(k\right)\right]}^{2} \end{array}$$ (10)

In Equation (10), $E_{x}$ is the energy of the nodule. The entropy of the nodule, as shown in Equation (11).


$$\begin{array}{l} H_{x}=-\sum_{k=0}^{L-1}H\left(k\right)\log H\left(k\right) \end{array}$$ (11)

In Equation (11), $H_{x}$ is the entropy of the nodule. The resulting calcification of the nodule, as shown in Eq. (12)


$$\begin{array}{l} CA_{x}=\frac{\sum_{j=1}^{i}Count\left(I_{j}\left(x,y\right)T\right)}{A_{x}} \end{array}$$ (12)

In Equation (12), $CA_{x}$ is the degree of calcification, and the texture features were extracted using the contrast and uniformity information of the Gram matrix (GLOM) matrix. Since coarse texture has higher uniformity and lower contrast, the uniformity of nodules is greater than NN and the contrast is less than NN. contrast, as shown in Equation (13).


$$\begin{array}{l} SL_{x}=\sum_{i=0}^{L-1}\sum_{j=0}^{L-1}{\left(i-j\right)}^{2}S_{d,\theta }\left(i,j\right) \end{array}$$ (13)

In Equation (13), $SL_{x}$ is the contrast of the nodule. And uniformity, as shown in Equation (14).


$$\begin{array}{l} SP_{x}=\sum_{i=0}^{L-1}\sum_{j=0}^{L-1}\frac{1}{1+{\left(i-j\right)}^{2}}S_{d,\theta }\left(i,j\right) \end{array}$$ (14)

**Figure 5.**
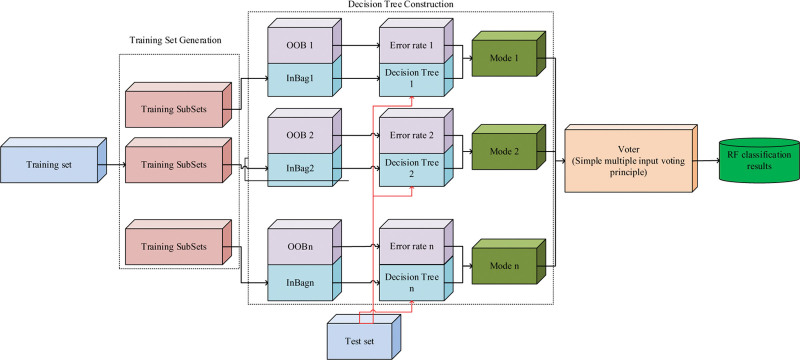
Flow chart of random forest classification.

In Equation (14), $SP_{x}$ is the degree of homogeneity. When facing larger datasets, random forests may be more prone to overfitting on larger datasets. A larger dataset provides more samples and variations, making the model more likely to remember the details of the training data rather than learning generalized patterns. To address this issue, strategies can be adopted, such as increasing the number of trees, limiting the maximum depth of each tree, or performing feature selection. On larger datasets, decision classification of random forests may be more robust. More samples and changes enable decision boundaries to better adapt to different data distributions. This means that random forests may be able to classify more accurately on larger datasets. It should be noted that different datasets and problems have different characteristics, so the specific situation may vary. When processing larger datasets, appropriate parameter tuning and evaluation are still needed to ensure the performance and generalization ability of the random forest model. Finally, a decision tree is used to test the test sample, and the output classification of each decision tree is aggregated, and the classification with the highest number of outputs from each decision tree is identified as the test sample according to the “majority vote” principle. The random forest model classification process is shown in Figure 5.

**Figure 6.**
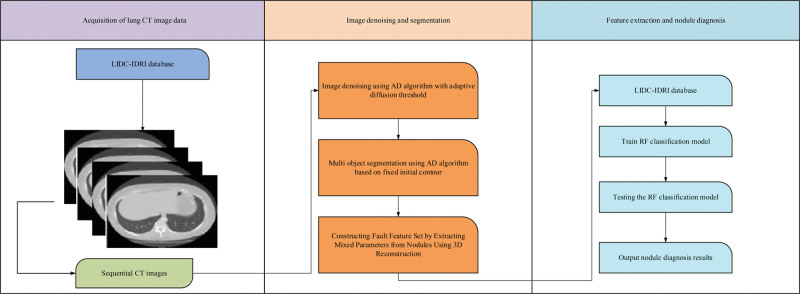
Diagnostic procedures of pulmonary nodules.

In Figure 5, the performance metrics of random forest are Generalization ability (Ga), Generalization Error (Ge). ga refers to the predictive ability of the network model obtained from a finite sample over the domain of other variables. ge is a metric reflecting the generalization ability, and the magnitude of the value is inversely proportional to the ML performance. The flow of the LN diagnosis method based on automatic image segmentation of geometric active contours and random forest is shown in Figure 6.

In Figure 6, firstly, the lung CT images were obtained using the LIDC-IDRI database; based on this, a multi-objective segmentation of the LN was achieved using non-homogeneous, non-homogeneous and non-homogeneous methods in combination with the existing active geometric contour information to obtain the LN candidate regions and obtain the 3D hybrid features; finally, the RF classifier method was used to detect and diagnosis.

## 4. Analysis of diagnostic models incorporating ML in LN images

By building an SVM diagnostic model, an LR diagnostic model, and a feature extraction model incorporated into a random forest, the feature vectors are obtained and the next step is to process the sequences made up of the feature vectors. Building a new diagnostic model helps to extract the information from the sequences, which is subsequently fused with being able to be stitched together to form a complete model.

### 4.1. Diagnostic model analysis of LN images incorporating SVM

**Figure 7.**
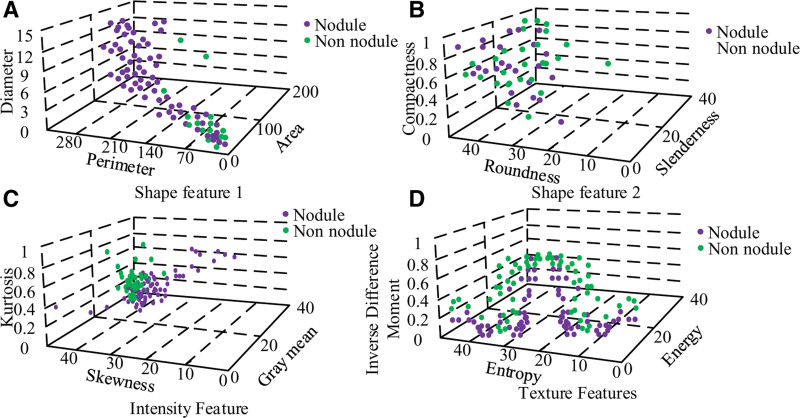
Differences in feature space.

Using multiple datasets, including the Limph Node Metastases (LNM) dataset, The Cancer Imaging Archive (TCIA) dataset, and the Medical Segmentation Decathlon (MSD) dataset, to combine the training of SVM models for LN image diagnostic performance. Although there may not be much improvement in the performance of the model from the training indicators, the performance of the model will be better on datasets of different models of machines or different film sources. This indicates that the robustness and stability of the model have been improved through supplementary training. By combining multiple datasets for training, the model can better adapt to different data sources and device differences. It is proposed to select 90 CT images from the LIDC database and to analyze 70 images, each containing about 300 images, for a total of 19,000 images. The pre-defined storage image format is DICOM, which is used to create, transfer and store medical images and report data, and to define the data dictionary, data structure, file format, client and server services, workflow, image compression and other transactions. The feature extraction process extracted a total of 17 features from 3 categories of features with the distribution values shown in Figure 7.

In Figure 7, for the characteristics of high-dimensional mixed features, this project proposes to use the LDA method to reduce their dimensionality, i.e. feature selection, in order to reduce the computational complexity of the classifier, remove redundant noise and improve the accuracy of the classifier. A matrix of 1216 nodes and 3407 non-nodes, consisting of the original 17 features, is dimensionalized to obtain a node of 17 × 1120 and a non-node of 17 × 2980. a feature-based classification method is proposed. We now use a complete high-dimensional mixed feature dataset as the benchmark dataset to address the noise and nonlinearity issues in high-dimensional mixed features. Generate a noisy dataset from this dataset. Select SVM as the benchmark classifier for subsequent control experiments. Train the classifier using a complete high-dimensional mixed feature dataset and test its performance on the test set. Train the classifier with a noisy dataset and test its performance on the test set. Apply a feature selection method to select a subset of features from the original dataset. Train the classifier using the selected feature subset and test its performance on the test set. Train the classifier with the same feature subset as the noise dataset and test its performance on the test set. Based on the experimental results, discuss how noise and nonlinearity in high-dimensional mixed features affect the performance of classifiers. To this end, the LDA method for dimensionality reduction of the hybrid features is necessary to improve the learning efficiency of the SVM. To verify the high performance of the SVM classifier, 3 sets of samples were used as experimental data to compare the naive Bias, KNN and SVM classifiers. The performance of naive Bias, KNN and SVM was tested by comparing the 3 sample sets. This is shown in Table 1.

**Table 1:** Performance comparison of different types of support vector machines.

Classifiers	Training/testing	Accuracy(%)	Sensitivity (%)	Specificity(%)
LDA based SVM	20/80	77.60	75.40	77.80
	40/60	78.50	76.30	76.50
	50/50	82.54	83.30	81.30
	60/40	86.46	85.50	83.25
	80/20	89.88	88.76	87.60
Rule-based SVM	20/80	84.40	85.30	83.20
	40/60	85.54	86.70	85.30
	50/50	88.30	89.30	87.50
	60/40	90.10	92.50	88.00
	80/20	93.40	94.50	89.00
LDA & rule Based SVM	20/80	87.90	90.20	86.40
	40/60	86.70	88.65	86.63
	50/50	90.30	93.30	89.10
	60/40	94.50	94.36	90.50
	80/20	98.96	98.76	96.68

LDA = linear discriminant analysis, SVM = support vector machine.

**Figure 8.**
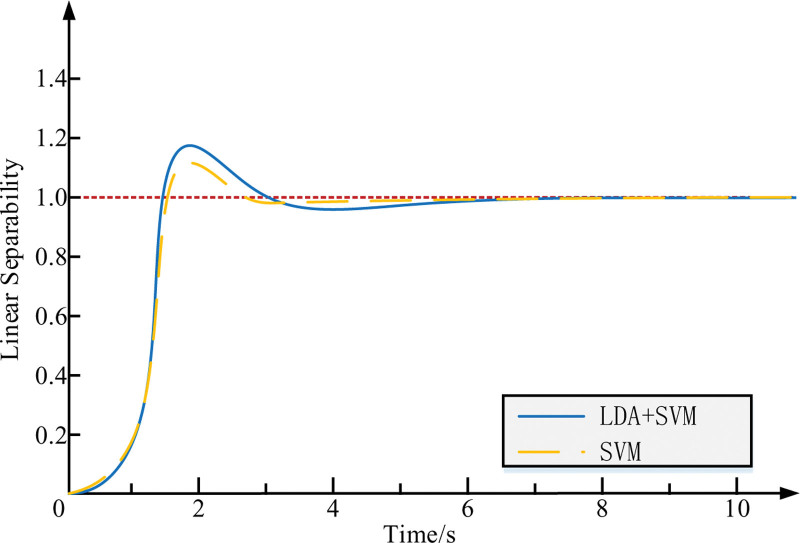
The ablation experiment of LDA + SVM and SVM. LDA = linear discriminant analysis, SVM = support vector machine.

In Table 1, the Bias classifier is based on joint probabilities and is easy to learn and train, but the performance of the classifier is not good for data with mixed characteristics. the KNN classifier achieves good classification results but suffers from being computationally intensive and not handling samples well. To make the results fairer, each result was performed on the same samples with and without knots. Compared with traditional classification methods, SVM classification methods have significant advantages in terms of accuracy, sensitivity and specificity. The ablation experiment of LDA + SVM and SVM, As shown in Figure 8.

In Figure 8, In our ablation experiment, we focused on the performance of LDA + SVM and SVM. During the experimental period of 0 to 10 seconds, LDA + SVM achieved a linear separability level of 1.2 at 2 seconds. This indicates that after using LDA for dimensionality reduction processing, SVM can more effectively find decision boundaries, thereby improving the accuracy of classification. At the same time, SVM achieved a linear separability level of 1.1 at 2 seconds within the same time range (0–10 seconds). Although it still has a certain degree of linear separability, its performance is slightly inferior to LDA + SVM. This ablation experiment demonstrates the importance of LDA in enhancing the linear separability of SVM, which helps us understand the advantages and limitations of LDA + SVM.

### 4.2. LN image construction analysis incorporating LR

The Peak Signal Noise Ratio (PSNR) and Figure of Merit (FOM) are 2 evaluation metrics used to evaluate their effectiveness. PSNR measures the approximation of the denoised CT image to the original image, the larger the value, the closer the denoised image is to the original image. This is shown in Table 2.

**Table 2:** Comparison of evaluation indicators for CT lung image denoising algorithms.

Index	Legend	Gaussian filter	PM model	Catte model	COM-AD
PSNR	1	25.2134	27.0735	28.1854	29.3377
	2	25.2739	27.1615	28.1983	29.4090
	3	25.2531	27.1085	28.2547	29.7487
	4	25.3020	27.2575	28.3521	29.1497
	5	25.4195	27.3241	28.4135	29.8855
FOM	1	0.8131	0.8894	0.8670	0.9014
	2	0.8074	0.8935	0.8795	0.9255
	3	0.8207	0.8977	0.8648	0.9241
	4	0.8197	0.8865	0.8724	0.9300
	5	0.8201	0.8923	0.8743	0.9379

**Figure 9.**
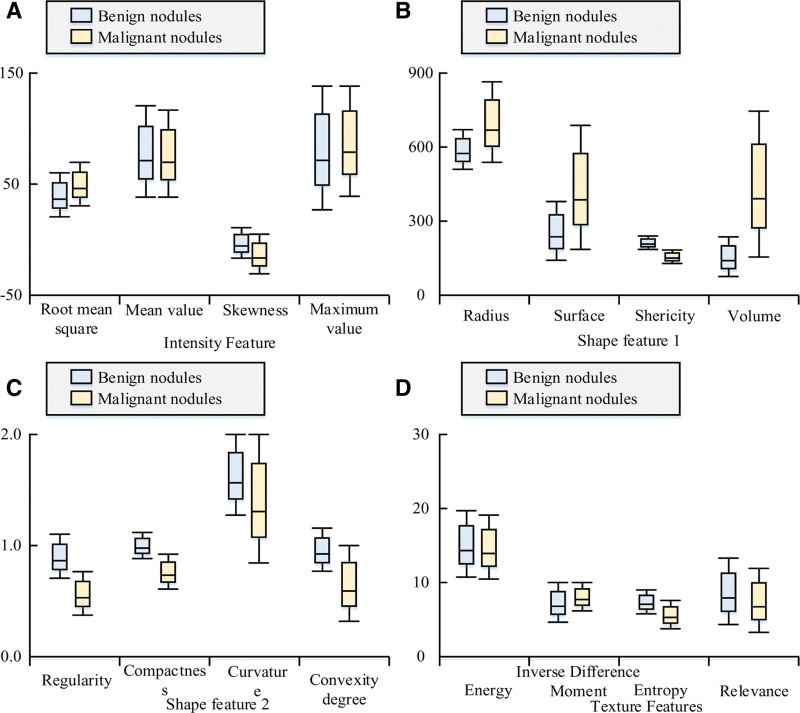
Extraction of feature box graphs.

In Table 2, the Catte model has better performance than the PM model in terms of PSNR values. However, the Catte model has an average result on the FOM, indicating that the Catte model has the problem of unclear boundaries and details. The comparative analysis of the 2 methods concluded that the COM-AD method has better results than the other methods in maintaining image edges and details, while the Gaussian method fails to achieve good results. To verify the quality of the 3D feature extraction, the extracted feature box plots, as shown in Figure 9.

In Figure 9, there is no significant difference between benign and malignant nodules in Figure 9A and D, and both features contain a small number of abnormal values in both. The distribution of median values shows that the gray-scale and textured data are symmetrical. The morphology of the benign and malignant tumors in the subplots of Figure 9B and C is very different and this difference can be seen in the box plots. Malignant tumors are larger in diameter, surface area and volume, and less spherical, regular, compact and bulging than benign tumors. Benign tumors are distinguished from cancerous nodules mainly by curvature characteristics.

### 4.3. Analysis of LN3D feature extraction and classifier construction incorporating random forest

**Figure 10.**
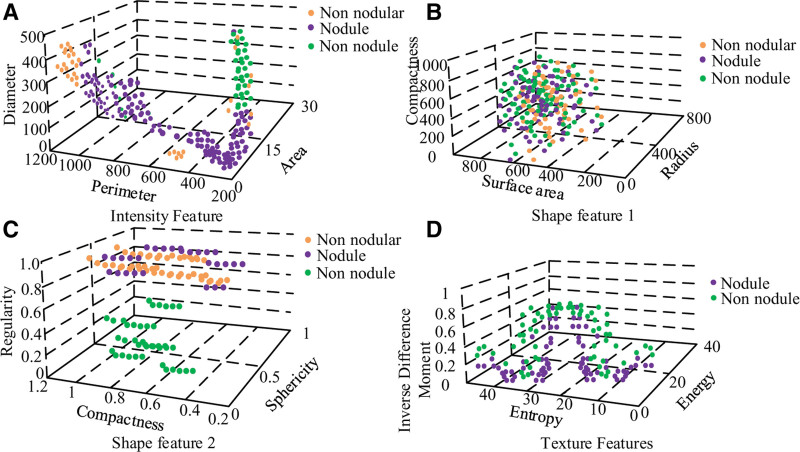
Extracting feature scatter maps.

Combining graph denoising with image segmentation, candidates for LN were obtained using automatic segmentation. In the feature extraction process, a total of 1260 features were extracted to form a 25 × 400 sample set of BN features, a 25 × 200 sample set of malignant nodules and a 25 × 660 sample set of NNs. A scatter plot of the extracted features for the 3 types of samples was plotted, as shown in Figure 10.

**Figure 11.**
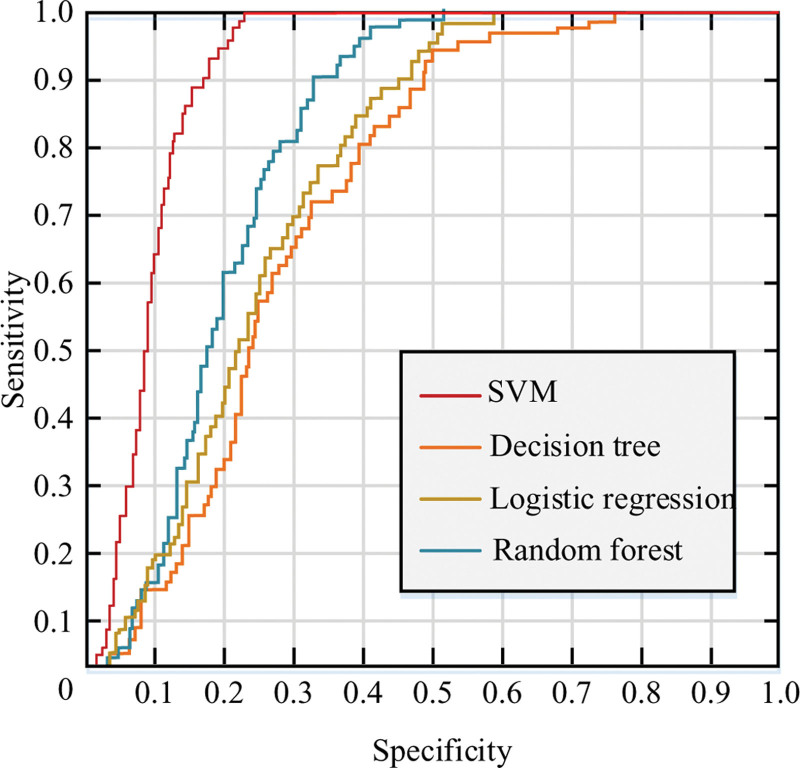
Receiver operating characteristic comparison.

In Figure 10, malignant nodules are easily distinguished from the other 2 types as seen in Figure 9B, where some of the nodule-free samples are submerged in the BN samples, and further classification of them is needed. The shape features in Figure 9C also clearly distinguish malignant nodules from BN, while the information on NN is mixed between the 2 categories. The diagnosis of the 3 features of Figure 9C and D, which are all non-linear and non-separable due to the scatter plot, requires an efficient classifier. The shot factors were compared using SVM and LR classifiers, where the SVM uses the kernel function of RBF and optimizes the parameters by 5 cross-checks, ultimately on the same test samples. The experimental results for plotting the ROC curves are shown in Figure 11.

**Figure 12.**
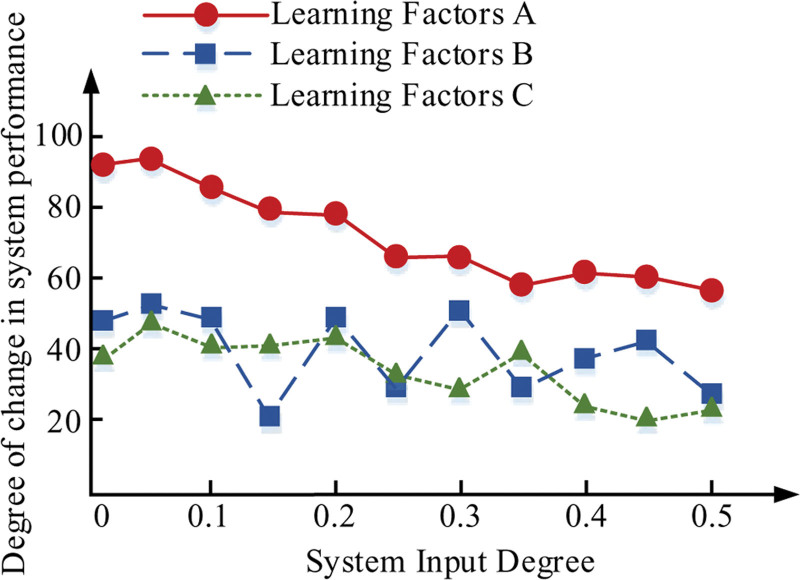
Robustness of data changes.

In Figure 11, 2 different training samples were used: one was a manually segmented LN image from a lung CT image, and the other was an LN image extracted from an LN disease teaching video downloaded from the Internet. The corresponding regions under the curve (AUC) were calculated from the curves as SVM (AUC = 0.917); RF (AUC = 0.717); LR (AUC = 0.652); and DT (AUC = [0.663]) respectively. Table 3 shows that SVM has the best performance among different networks. The robustness against data changes is shown in Figure 12.

**Table 3:** The performance metrics of different models.

Model	Precision	Recall	F1 score
SVM	0.893	0.826	0.845
LR	0.675	0.823	0.765
RF	0.465	0.865	0.759
DT	0.645	0.807	0.716

LR = logistic regression, SVM = support vector machine.

In Figure 12, the ordinary LR classifier may not achieve the ideal performance when dealing with various noises and uncertainties. However, by optimizing the LR algorithm using Particle Sd Optimization (PSO), we found a significant improvement in classification performance. Here, we treat the performance of the traditional LR classifier as a baseline, with a score of 68. However, the performance of the LR classifier improved to 85 when using PSO optimization. Moreover, when we performed further testing of robustness to the PSO-LR algorithm, its performance remained excellent even in the face of complex datasets, reaching a score of 94. In specific complex situations, such as the sudden increase in the noise level in the data, or the sudden change of the correlation between features, the PSO-LR algorithm is still able to maintain relatively high performance. At these specific test points, they scored 76,75,81 and 84, all of which outperform LR classifiers not optimized using PSO. Overall, after averaging the classification error rates across all test cases, the traditional LR classifier, and PSO-LR classifier with further robust examination were 24,15 and 9, respectively. Specifically, the PSO-LR classifier had the smallest error, with 64% higher accuracy compared to the original LR algorithm and 45% improvement compared to the PSO-optimized LR algorithm. The robustness assessment method used evaluates the performance of the classifier when dealing with data with uncertainty and noise. Specifically, the factors considered include the noise level in the data, the correlation between features, and the complexity of the data.

## 5. Discussion

The traditional diagnosis of pulmonary nodules uses routine lung CT scans of the examination population, which are then manually read by radiologists. Although this traditional diagnostic method is classic, the efficiency of manual film reading is low, the diagnostic results may vary from person to person, and the rates of misdiagnosis and missed diagnosis are relatively high. Compared to this, machine learning-based imaging diagnosis methods have the advantages of efficiency and accuracy. Machine learning can automatically identify and evaluate pulmonary nodules through a Big data training diagnosis model. The diagnostic results are more objective, avoiding possible differences when manually reading the film. Scholars such as Y F Li explored the differential value of CT texture features for benign and malignant pulmonary nodules and their generalization ability on independent datasets. The AUC, coincidence rate, sensitivity, specificity, positive predictive value (PPV) and negative predictive value (NPV) obtained by cross-validation of the optimal model in the training set were 0.892, 0.859, 0.788, 0.876, 0.492 and 0.964, respectively. After Feature selection, a total of 17 iconographic features were included in the classification diagnosis model of benign and malignant pulmonary nodules. In the validation set, the AUC, coincidence rate, sensitivity, specificity, PPV and NPV are 0.765, 0.745, 0.800, 0.700, 0.689, and 0.808, respectively. This study results, along with the results of this experiment, validate the efficient and accurate advantages of machine learning-based imaging diagnosis methods.^[18,19]^ Scholars such as Z Wang used machine learning-based classification and judgment algorithms to establish a staging model for chronic obstructive pulmonary disease (COPD), improving the accuracy of diagnosis and staging. The data is imbalanced, although stratified proportional sampling is used, However, the accuracy of SVM for such data is higher at 85.26%. This indicates that the model provided by machine learning can provide a more accurate classification basis for the staging of chronic obstructive pulmonary disease. This study also indirectly proves the effectiveness of machine learning in the problem of pulmonary nodules.^[20]^ In addition, a major advantage of machine learning is its iterative learning ability, which can gradually improve its recognition and classification accuracy for pulmonary nodule images. However, machine learning-based diagnostic methods also face some challenges. Comparing the advantages and disadvantages of traditional methods with new research methods will bring innovative concepts and perspectives to the intelligent diagnosis of pulmonary nodules, innovate diagnostic methods for pulmonary nodules, and strive for more survival opportunities in the future.

This study is characterized by delving into the PSO algorithm based on SVM, combined with the random forest algorithm, enabling the proposed machine learning algorithm to assist medical personnel in diagnosing patients’ conditions through intelligent imaging diagnostics. By extracting hybrid features that include various characteristics such as grayscale, shape, and texture, it enhances the representation of the candidate nodes’ feature information. In terms of node identification, this paper overcomes the shortcomings of traditional single classifiers and proposes a hybrid classifier based on Support Vector Machine combined with rules, providing unique insights into the relationships between imaging features, and between features and disease classification. This is more conducive to doctors’ diagnosis of different categories of pulmonary nodules.

## 6. Conclusion

An ID-based LNID model is created based on the ML technique using the LN as the research object from a diagnostic standpoint. Based on this, the learner knowledge is modeled using the optimized ML methodology, and the matching ID model is constructed by combining better deep learning and adaptive learning techniques. According to experiments, benign and malignant tumors have highly different morphological characteristics, which are clearly visible on the box plot. Malignant tumors are less spherical, regular, compact, and bulging than benign tumors, and they are greater in diameter, surface area, and volume. Curvature characteristics are the main way that benign tumors and malignant nodules can be identified. Morphological characteristics can confuse messages about asexual malignancies across the 2 categories and help identify tumors from benign tumors. In terms of PSNR values, the Carter model outperforms the PM model. The Catte model, however, performs poorly on the FOM, showing that it has ambiguous borders and specifics. To categorize these 3 features, an effective classifier is required because they are clearly non-linear and non-separable. The comparable AUC curves for SVM, RF, LR value, and interferon are 0.917, 0.717, 0.652, and 0.516, respectively. Despite the positive research findings, there are still certain communication and immersion issues that need to be addressed in future studies because to the limited domain knowledge that corresponds to LN pictures. LN is a prevalent illness in LT. As a result, it is crucial to create an ML-based LNID model on this foundation in order to help with the medical imaging diagnosis issue.

## Author contributions

**Data curation:** Lilei Zhuang.

**Formal analysis:** Sheng Hu.

**Investigation:** Li Sun.

**Methodology:** Yangxiang Liu.

**Supervision:** Zhengwei Dou.

**Writing – original draft:** Miaomiao Li.

**Writing – review & editing:** Miaomiao Li, Tao Jang.
